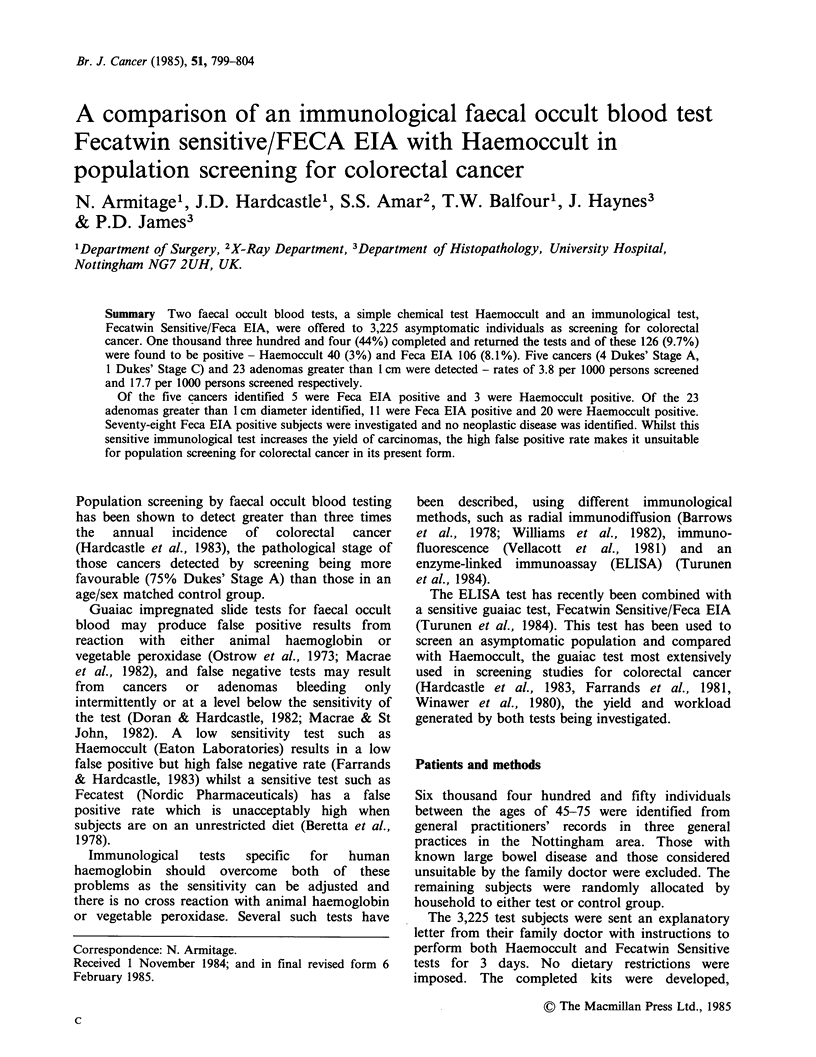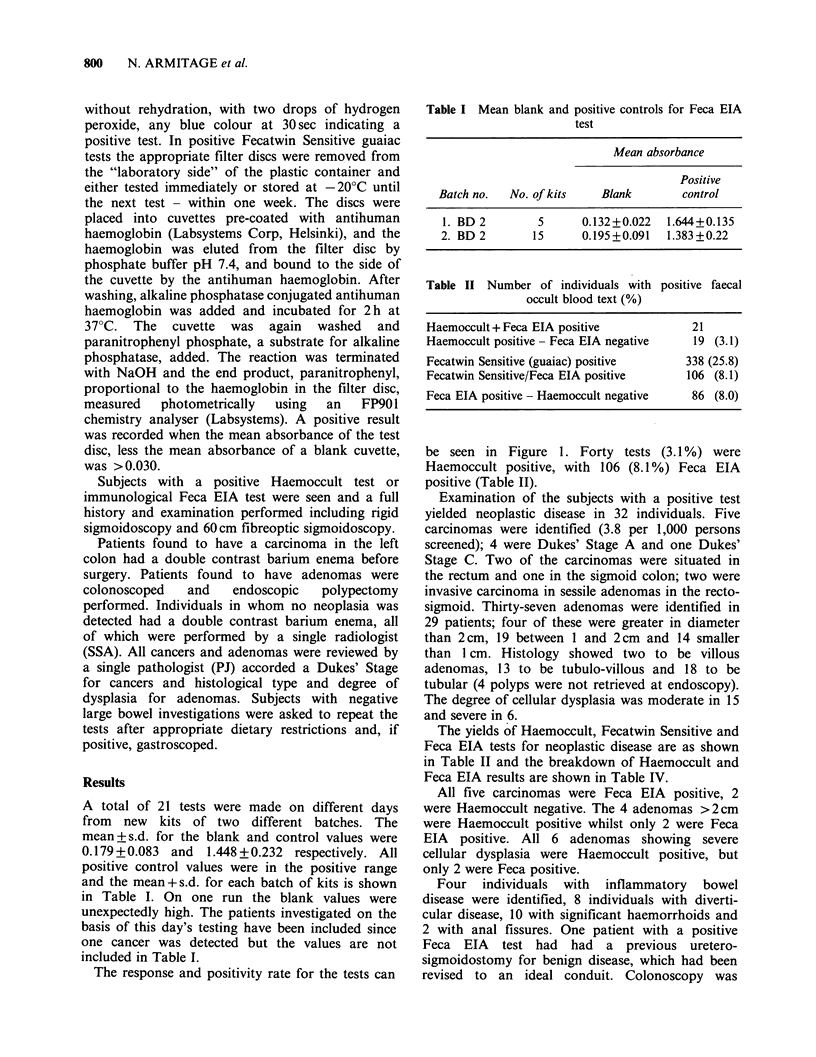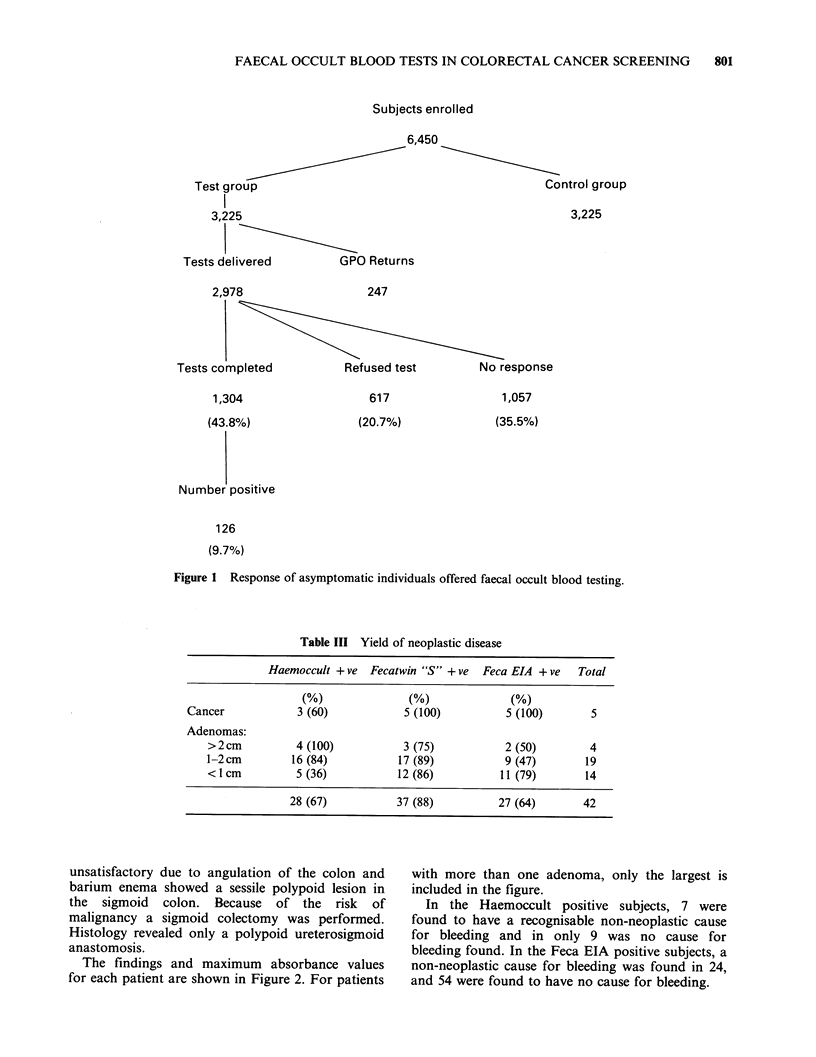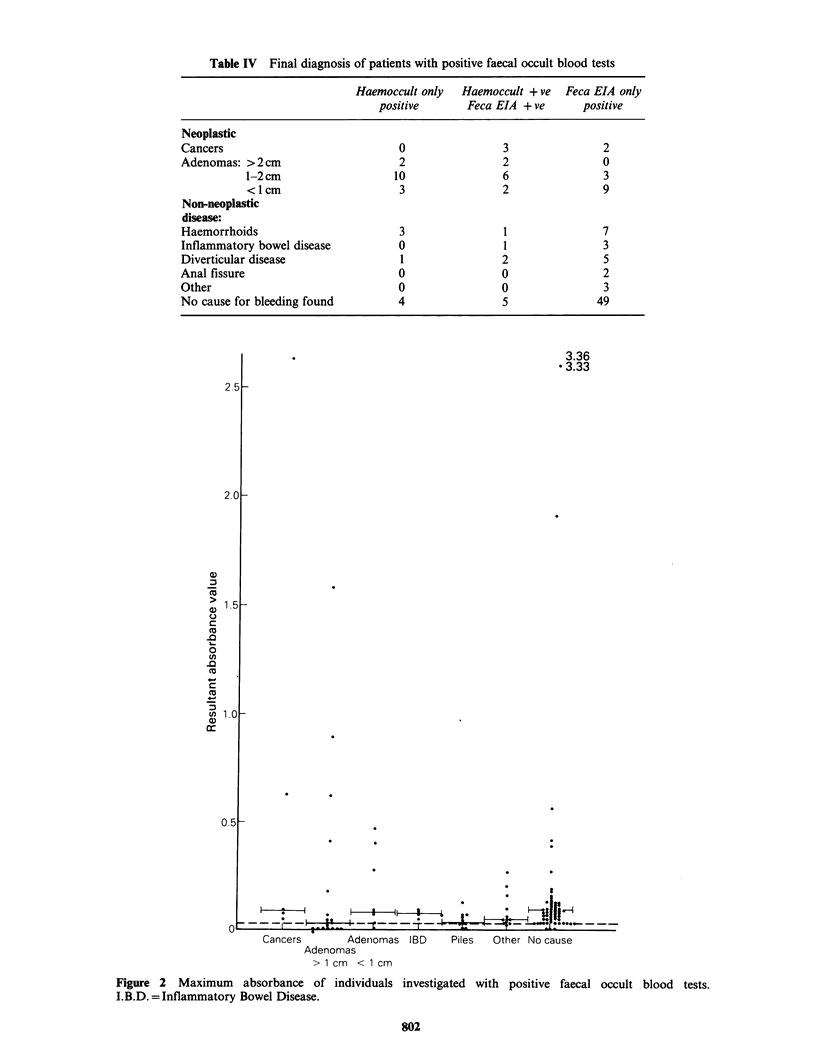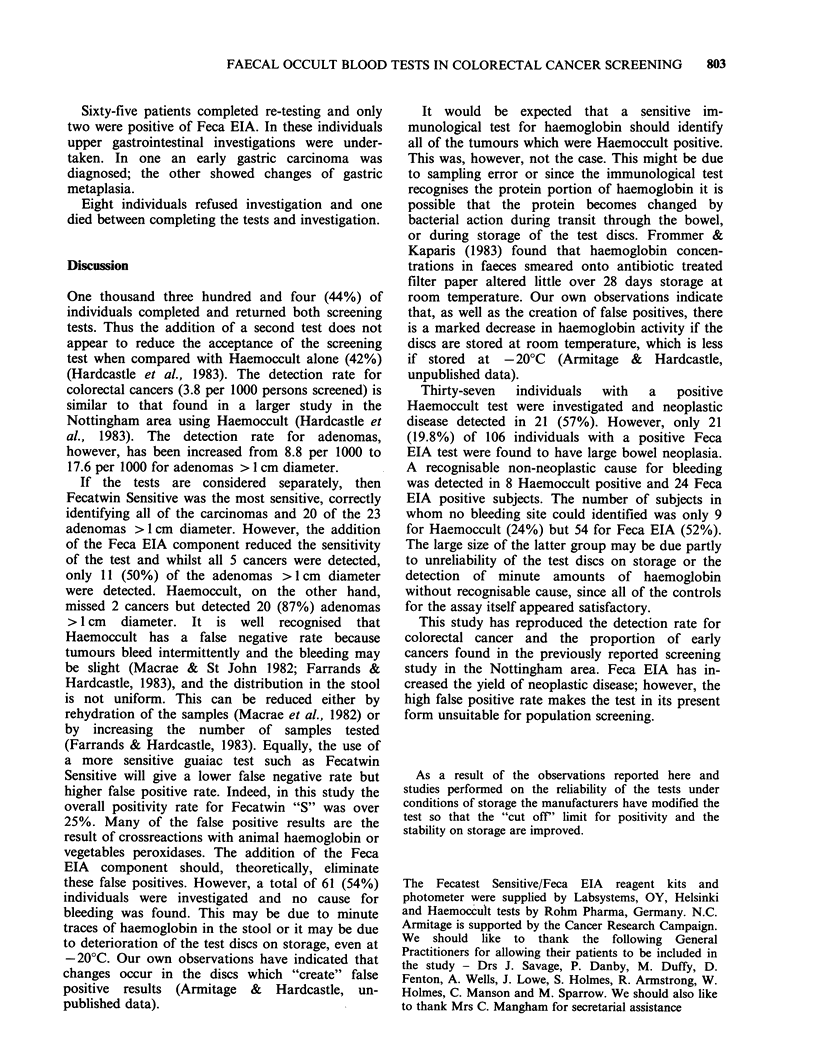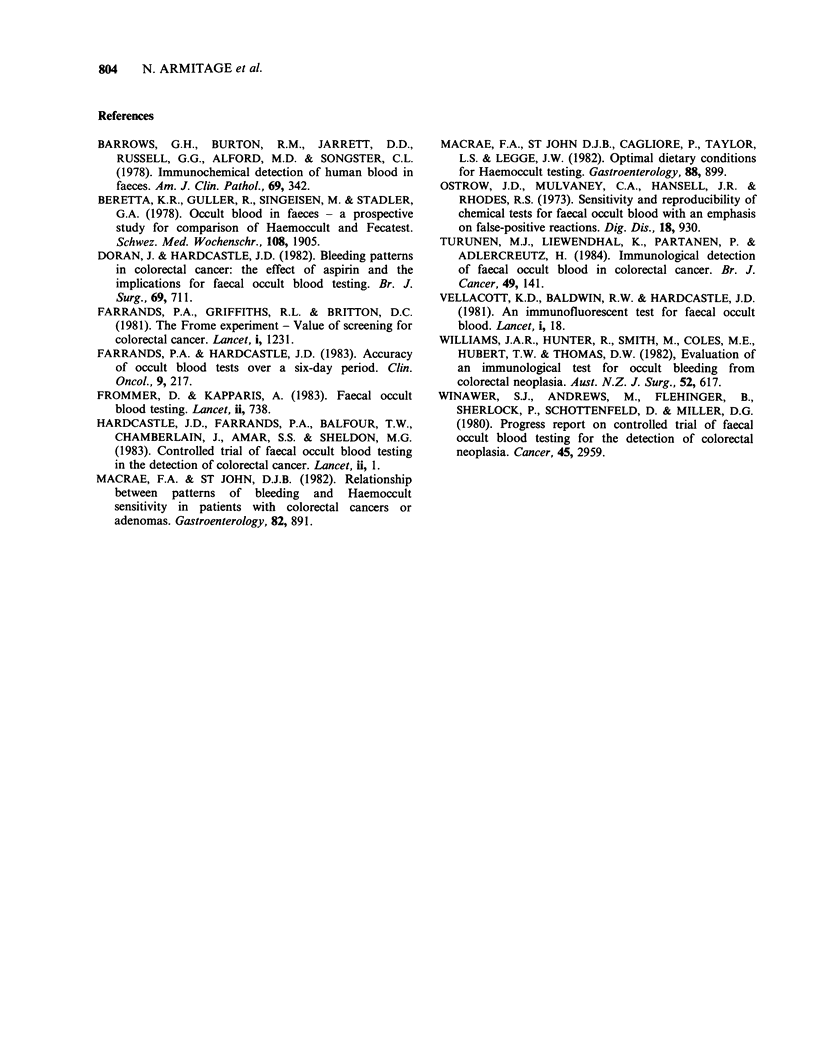# A comparison of an immunological faecal occult blood test Fecatwin sensitive/FECA EIA with Haemoccult in population screening for colorectal cancer.

**DOI:** 10.1038/bjc.1985.124

**Published:** 1985-06

**Authors:** N. Armitage, J. D. Hardcastle, S. S. Amar, T. W. Balfour, J. Haynes, P. D. James

## Abstract

Two faecal occult blood tests, a simple chemical test Haemoccult and an immunological test, Fecatwin Sensitive/Feca EIA, were offered to 3,225 asymptomatic individuals as screening for colorectal cancer. One thousand three hundred and four (44%) completed and returned the tests and of these 126 (9.7%) were found to be positive - Haemoccult 40 (3%) and Feca EIA 106 (8.1%). Five cancers (4 Dukes' Stage A, 1 Dukes' Stage C) and 23 adenomas greater than 1 cm were detected - rates of 3.8 per 1000 persons screened and 17.7 per 1000 persons screened respectively. Of the five cancers identified 5 were Feca EIA positive and 3 were Haemoccult positive. Of the 23 adenomas greater than 1 cm diameter identified, J1 were Feca EIA positive and 20 were Haemoccult positive. Seventy-eight Feca EIA positive subjects were investigated and no neoplastic disease was identified. Whilst this sensitive immunological test increases the yield of carcinomas, the high false positive rate makes it unsuitable for population screening for colorectal cancer in its present form.


Br. J. Cancer (1985), 51, 799–804

### A comparison of an immunological faecal occult blood test Fecatwin sensitive/FECA EIA with Haemoccult in population screening for colorectal cancer

N. Armitage[1], J.D. Hardcastle[1], S.S. Amar[2], T.W. Balfour[1], J. Haynes[3] & P.D. James[3]

[1]*Department of Surgery,* [2]*X-Ray Department,* [3]*Department of Histopathology, University Hospital, Nottingham NG7 2UH, UK.*

**Summary** Two faecal occult blood tests, a simple chemical test Haemoccult and an immunological test, Fecatwin Sensitive/Feca EIA, were offered to 3,225 asymptomatic individuals as screening for colorectal cancer. One thousand three hundred and four (44%) completed and returned the tests and of these 126 (9.7%) were found to be positive – Haemoccult 40 (3%) and Feca EIA 106 (8.1%). Five cancers (4 Dukes' Stage A, 1 Dukes' Stage C) and 23 adenomas greater than 1 cm were detected – rates of 3.8 per 1000 persons screened and 17.7 per 1000 persons screened respectively.

Of the five cancers identified 5 were Feca EIA positive and 3 were Haemoccult positive. Of the 23 adenomas greater than 1 cm diameter identified, 11 were Feca EIA positive and 20 were Haemoccult positive. Seventy-eight Feca EIA positive subjects were investigated and no neoplastic disease was identified. Whilst this sensitive immunological test increases the yield of carcinomas, the high false positive rate makes it unsuitable for population screening for colorectal cancer in its present form.

Population screening by faecal occult blood testing has been shown to detect greater than three times the annual incidence of colorectal cancer (Hardcastle *et al.*, 1983), the pathological stage of those cancers detected by screening being more favourable (75% Dukes' Stage A) than those in an age/sex matched control group.

Guaiac impregnated slide tests for faecal occult blood may produce false positive results from reaction with either animal haemoglobin or vegetable peroxidase (Ostrow *et al.*, 1973; Macrae *et al.*, 1982), and false negative tests may result from cancers or adenomas bleeding only intermittently or at a level below the sensitivity of the test (Doran & Hardcastle, 1982; Macrae & St John, 1982). A low sensitivity test such as Haemoccult (Eaton Laboratories) results in a low false positive but high false negative rate (Farrands & Hardcastle, 1983) whilst a sensitive test such as Fecatest (Nordic Pharmaceuticals) has a false positive rate which is unacceptably high when subjects are on an unrestricted diet (Beretta *et al.*, 1978).

Immunological tests specific for human haemoglobin should overcome both of these problems as the sensitivity can be adjusted and there is no cross reaction with animal haemoglobin or vegetable peroxidase. Several such tests have been described, using different immunological methods, such as radial immunodiffusion (Barrows *et al.*, 1978; Williams *et al.*, 1982), immunofluorescence (Vellacott *et al.*, 1981) and an enzyme-linked immunoassay (ELISA) (Turunen *et al.*, 1984).

The ELISA test has recently been combined with a sensitive guaiac test, Fecatwin Sensitive/Feca EIA (Turunen *et al.*, 1984). This test has been used to screen an asymptomatic population and compared with Haemoccult, the guaiac test most extensively used in screening studies for colorectal cancer (Hardcastle *et al.*, 1983, Farrands *et al.*, 1981, Winawer *et al.*, 1980), the yield and workload generated by both tests being investigated.

### Patients and methods

Six thousand four hundred and fifty individuals between the ages of 45–75 were identified from general practitioners' records in three general practices in the Nottingham area. Those with known large bowel disease and those considered unsuitable by the family doctor were excluded. The remaining subjects were randomly allocated by household to either test or control group.

The 3,225 test subjects were sent an explanatory letter from their family doctor with instructions to perform both Haemoccult and Fecatwin Sensitive tests for 3 days. No dietary restrictions were imposed. The completed kits were developed,

Correspondence: N. Armitage.
Received 1 November 1984; and in final revised form 6 February 1985.

© The Macmillan Press Ltd., 1985

without rehydration, with two drops of hydrogen peroxide, any blue colour at 30 sec indicating a positive test. In positive Fecatwin Sensitive guaiac tests the appropriate filter discs were removed from the "laboratory side" of the plastic container and either tested immediately or stored at −20°C until the next test – within one week. The discs were placed into cuvettes pre-coated with antihuman haemoglobin (Labsystems Corp, Helsinki), and the haemoglobin was eluted from the filter disc by phosphate buffer pH 7.4, and bound to the side of the cuvette by the antihuman haemoglobin. After washing, alkaline phosphatase conjugated antihuman haemoglobin was added and incubated for 2 h at 37°C. The cuvette was again washed and paranitrophenyl phosphate, a substrate for alkaline phosphatase, added. The reaction was terminated with NaOH and the end product, paranitrophenyl, proportional to the haemoglobin in the filter disc, measured photometrically using an FP901 chemistry analyser (Labsystems). A positive result was recorded when the mean absorbance of the test disc, less the mean absorbance of a blank cuvette, was >0.030.

Subjects with a positive Haemoccult test or immunological Feca EIA test were seen and a full history and examination performed including rigid sigmoidoscopy and 60 cm fibreoptic sigmoidoscopy.

Patients found to have a carcinoma in the left colon had a double contrast barium enema before surgery. Patients found to have adenomas were colonoscoped and endoscopic polypectomy performed. Individuals in whom no neoplasia was detected had a double contrast barium enema, all of which were performed by a single radiologist (SSA). All cancers and adenomas were reviewed by a single pathologist (PJ) accorded a Dukes' Stage for cancers and histological type and degree of dysplasia for adenomas. Subjects with negative large bowel investigations were asked to repeat the tests after appropriate dietary restrictions and, if positive, gastroscoped.

### Results

A total of 21 tests were made on different days from new kits of two different batches. The mean ± s.d. for the blank and control values were 0.179 ± 0.083 and 1.448 ± 0.232 respectively. All positive control values were in the positive range and the mean + s.d. for each batch of kits is shown in Table I. On one run the blank values were unexpectedly high. The patients investigated on the basis of this day's testing have been included since one cancer was detected but the values are not included in Table I.

The response and positivity rate for the tests can be seen in Figure 1. Forty tests (3.1%) were Haemoccult positive, with 106 (8.1%) Feca EIA positive (Table II).

**Table I** Mean blank and positive controls for Feca EIA test

| | | *Mean absorbance* | |
|---|---|---|---|
| *Batch no.* | *No. of kits* | *Blank* | *Positive control* |
| 1. BD 2 | 5 | 0.132 ± 0.022 | 1.644 ± 0.135 |
| 2. BD 2 | 15 | 0.195 ± 0.091 | 1.383 ± 0.22 |

**Table II** Number of individuals with positive faecal occult blood text (%)

| | |
|---|---|
| Haemoccult + Feca EIA positive | 21 |
| Haemoccult positive – Feca EIA negative | 19 (3.1) |
| Fecatwin Sensitive (guaiac) positive | 338 (25.8) |
| Fecatwin Sensitive/Feca EIA positive | 106 (8.1) |
| Feca EIA positive – Haemoccult negative | 86 (8.0) |

Examination of the subjects with a positive test yielded neoplastic disease in 32 individuals. Five carcinomas were identified (3.8 per 1,000 persons screened); 4 were Dukes' Stage A and one Dukes' Stage C. Two of the carcinomas were situated in the rectum and one in the sigmoid colon; two were invasive carcinoma in sessile adenomas in the recto-sigmoid. Thirty-seven adenomas were identified in 29 patients; four of these were greater in diameter than 2 cm, 19 between 1 and 2 cm and 14 smaller than 1 cm. Histology showed two to be villous adenomas, 13 to be tubulo-villous and 18 to be tubular (4 polyps were not retrieved at endoscopy). The degree of cellular dysplasia was moderate in 15 and severe in 6.

The yields of Haemoccult, Fecatwin Sensitive and Feca EIA tests for neoplastic disease are as shown in Table II and the breakdown of Haemoccult and Feca EIA results are shown in Table IV.

All five carcinomas were Feca EIA positive, 2 were Haemoccult negative. The 4 adenomas >2 cm were Haemoccult positive whilst only 2 were Feca EIA positive. All 6 adenomas showing severe cellular dysplasia were Haemoccult positive, but only 2 were Feca positive.

Four individuals with inflammatory bowel disease were identified, 8 individuals with diverticular disease, 10 with significant haemorrhoids and 2 with anal fissures. One patient with a positive Feca EIA test had had a previous uretero-sigmoidostomy for benign disease, which had been revised to an ideal conduit. Colonoscopy was

**Figure 1** Response of asymptomatic individuals offered faecal occult blood testing.

**Table III** Yield of neoplastic disease

| | *Haemoccult +ve* | *Fecatwin "S" +ve* | *Feca EIA +ve* | *Total* |
|---|---|---|---|---|
| | (%) | (%) | (%) | |
| Cancer | 3 (60) | 5 (100) | 5 (100) | 5 |
| Adenomas: | | | | |
| >2 cm | 4 (100) | 3 (75) | 2 (50) | 4 |
| 1–2 cm | 16 (84) | 17 (89) | 9 (47) | 19 |
| <1 cm | 5 (36) | 12 (86) | 11 (79) | 14 |
| | 28 (67) | 37 (88) | 27 (64) | 42 |

unsatisfactory due to angulation of the colon and barium enema showed a sessile polypoid lesion in the sigmoid colon. Because of the risk of malignancy a sigmoid colectomy was performed. Histology revealed only a polypoid ureterosigmoid anastomosis.

The findings and maximum absorbance values for each patient are shown in Figure 2. For patients with more than one adenoma, only the largest is included in the figure.

In the Haemoccult positive subjects, 7 were found to have a recognisable non-neoplastic cause for bleeding and in only 9 was no cause for bleeding found. In the Feca EIA positive subjects, a non-neoplastic cause for bleeding was found in 24, and 54 were found to have no cause for bleeding.

**Table IV** Final diagnosis of patients with positive faecal occult blood tests

| | *Haemoccult only positive* | *Haemoccult +ve Feca EIA +ve* | *Feca EIA only positive* |
|---|---|---|---|
| **Neoplastic** | | | |
| Cancers | 0 | 3 | 2 |
| Adenomas: >2 cm | 2 | 2 | 0 |
| 1–2 cm | 10 | 6 | 3 |
| <1 cm | 3 | 2 | 9 |
| **Non-neoplastic disease:** | | | |
| Haemorrhoids | 3 | 1 | 7 |
| Inflammatory bowel disease | 0 | 1 | 3 |
| Diverticular disease | 1 | 2 | 5 |
| Anal fissure | 0 | 0 | 2 |
| Other | 0 | 0 | 3 |
| No cause for bleeding found | 4 | 5 | 49 |

**Figure 2** Maximum absorbance of individuals investigated with positive faecal occult blood tests. I.B.D. = Inflammatory Bowel Disease.

Sixty-five patients completed re-testing and only two were positive of Feca EIA. In these individuals upper gastrointestinal investigations were undertaken. In one an early gastric carcinoma was diagnosed; the other showed changes of gastric metaplasia.

Eight individuals refused investigation and one died between completing the tests and investigation.

### Discussion

One thousand three hundred and four (44%) of individuals completed and returned both screening tests. Thus the addition of a second test does not appear to reduce the acceptance of the screening test when compared with Haemoccult alone (42%) (Hardcastle *et al.*, 1983). The detection rate for colorectal cancers (3.8 per 1000 persons screened) is similar to that found in a larger study in the Nottingham area using Haemoccult (Hardcastle *et al.*, 1983). The detection rate for adenomas, however, has been increased from 8.8 per 1000 to 17.6 per 1000 for adenomas >1 cm diameter.

If the tests are considered separately, then Fecatwin Sensitive was the most sensitive, correctly identifying all of the carcinomas and 20 of the 23 adenomas >1 cm diameter. However, the addition of the Feca EIA component reduced the sensitivity of the test and whilst all 5 cancers were detected, only 11 (50%) of the adenomas >1 cm diameter were detected. Haemoccult, on the other hand, missed 2 cancers but detected 20 (87%) adenomas >1 cm diameter. It is well recognised that Haemoccult has a false negative rate because tumours bleed intermittently and the bleeding may be slight (Macrae & St John 1982; Farrands & Hardcastle, 1983), and the distribution in the stool is not uniform. This can be reduced either by rehydration of the samples (Macrae *et al.*, 1982) or by increasing the number of samples tested (Farrands & Hardcastle, 1983). Equally, the use of a more sensitive guaiac test such as Fecatwin Sensitive will give a lower false negative rate but higher false positive rate. Indeed, in this study the overall positivity rate for Fecatwin "S" was over 25%. Many of the false positive results are the result of crossreactions with animal haemoglobin or vegetables peroxidases. The addition of the Feca EIA component should, theoretically, eliminate these false positives. However, a total of 61 (54%) individuals were investigated and no cause for bleeding was found. This may be due to minute traces of haemoglobin in the stool or it may be due to deterioration of the test discs on storage, even at −20°C. Our own observations have indicated that changes occur in the discs which "create" false positive results (Armitage & Hardcastle, unpublished data).

It would be expected that a sensitive immunological test for haemoglobin should identify all of the tumours which were Haemoccult positive. This was, however, not the case. This might be due to sampling error or since the immunological test recognises the protein portion of haemoglobin it is possible that the protein becomes changed by bacterial action during transit through the bowel, or during storage of the test discs. Frommer & Kaparis (1983) found that haemoglobin concentrations in faeces smeared onto antibiotic treated filter paper altered little over 28 days storage at room temperature. Our own observations indicate that, as well as the creation of false positives, there is a marked decrease in haemoglobin activity if the discs are stored at room temperature, which is less if stored at −20°C (Armitage & Hardcastle, unpublished data).

Thirty-seven individuals with a positive Haemoccult test were investigated and neoplastic disease detected in 21 (57%). However, only 21 (19.8%) of 106 individuals with a positive Feca EIA test were found to have large bowel neoplasia. A recognisable non-neoplastic cause for bleeding was detected in 8 Haemoccult positive and 24 Feca EIA positive subjects. The number of subjects in whom no bleeding site could identified was only 9 for Haemoccult (24%) but 54 for Feca EIA (52%). The large size of the latter group may be due partly to unreliability of the test discs on storage or the detection of minute amounts of haemoglobin without recognisable cause, since all of the controls for the assay itself appeared satisfactory.

This study has reproduced the detection rate for colorectal cancer and the proportion of early cancers found in the previously reported screening study in the Nottingham area. Feca EIA has increased the yield of neoplastic disease; however, the high false positive rate makes the test in its present form unsuitable for population screening.

As a result of the observations reported here and studies performed on the reliability of the tests under conditions of storage the manufacturers have modified the test so that the "cut off" limit for positivity and the stability on storage are improved.

The Fecatest Sensitive/Feca EIA reagent kits and photometer were supplied by Labsystems, OY, Helsinki and Haemoccult tests by Rohm Pharma, Germany. N.C. Armitage is supported by the Cancer Research Campaign. We should like to thank the following General Practitioners for allowing their patients to be included in the study – Drs J. Savage, P. Danby, M. Duffy, D. Fenton, A. Wells, J. Lowe, S. Holmes, R. Armstrong, W. Holmes, C. Manson and M. Sparrow. We should also like to thank Mrs C. Mangham for secretarial assistance

### References

BARROWS, G.H., BURTON, R.M., JARRETT, D.D., RUSSELL, G.G., ALFORD, M.D. & SONGSTER, C.L. (1978). Immunochemical detection of human blood in faeces. *Am. J. Clin. Pathol.*, **69**, 342.

BERETTA, K.R., GULLER, R., SINGEISEN, M. & STADLER, G.A. (1978). Occult blood in faeces – a prospective study for comparison of Haemoccult and Fecatest. *Schwez. Med. Wochenschr.*, **108**, 1905.

DORAN, J. & HARDCASTLE, J.D. (1982). Bleeding patterns in colorectal cancer: the effect of aspirin and the implications for faecal occult blood testing. *Br. J. Surg.*, **69**, 711.

FARRANDS, P.A., GRIFFITHS, R.L. & BRITTON, D.C. (1981). The Frome experiment – Value of screening for colorectal cancer. *Lancet*, **i**, 1231.

FARRANDS, P.A. & HARDCASTLE, J.D. (1983). Accuracy of occult blood tests over a six-day period. *Clin. Oncol.*, **9**, 217.

FROMMER, D. & KAPPARIS, A. (1983). Faecal occult blood testing. *Lancet*, **ii**, 738.

HARDCASTLE, J.D., FARRANDS, P.A., BALFOUR, T.W., CHAMBERLAIN, J., AMAR, S.S. & SHELDON, M.G. (1983). Controlled trial of faecal occult blood testing in the detection of colorectal cancer. *Lancet*, **ii**, 1.

MACRAE, F.A. & ST JOHN, D.J.B. (1982). Relationship between patterns of bleeding and Haemoccult sensitivity in patients with colorectal cancers or adenomas. *Gastroenterology*, **82**, 891.

MACRAE, F.A., ST JOHN D.J.B., CAGLIORE, P., TAYLOR, L.S. & LEGGE, J.W. (1982). Optimal dietary conditions for Haemoccult testing. *Gastroenterology*, **88**, 899.

OSTROW, J.D., MULVANEY, C.A., HANSELL, J.R. & RHODES, R.S. (1973). Sensitivity and reproducibility of chemical tests for faecal occult blood with an emphasis on false-positive reactions. *Dig. Dis.*, **18**, 930.

TURUNEN, M.J., LIEWENDHAL, K., PARTANEN, P. & ADLERCREUTZ, H. (1984). Immunological detection of faecal occult blood in colorectal cancer. *Br. J. Cancer*, **49**, 141.

VELLACOTT, K.D., BALDWIN, R.W. & HARDCASTLE, J.D. (1981). An immunofluorescent test for faecal occult blood. *Lancet*, **i**, 18.

WILLIAMS, J.A.R., HUNTER, R., SMITH, M., COLES, M.E., HUBERT, T.W. & THOMAS, D.W. (1982), Evaluation of an immunological test for occult bleeding from colorectal neoplasia. *Aust. N.Z. J. Surg.*, **52**, 617.

WINAWER, S.J., ANDREWS, M., FLEHINGER, B., SHERLOCK, P., SCHOTTENFELD, D. & MILLER, D.G. (1980). Progress report on controlled trial of faecal occult blood testing for the detection of colorectal neoplasia. *Cancer*, **45**, 2959.